# Improving transcriptome construction in non-model organisms: integrating manual and automated gene definition in *Emiliania huxleyi*

**DOI:** 10.1186/1471-2164-15-148

**Published:** 2014-02-22

**Authors:** Ester Feldmesser, Shilo Rosenwasser, Assaf Vardi, Shifra Ben-Dor

**Affiliations:** 1 Nancy and Stephen Grand Israel National Center for Personalized Medicine, Weizmann Institute of Science, Rehovot 76100, Israel; 2 Department of Plant Sciences, Weizmann Institute of Science, Rehovot 76100, Israel; 3 Department of Biological Services, Weizmann Institute of Science, Rehovot 76100, Israel

**Keywords:** RNAseq, Non-model organism, Transcriptome assembly, Manual curation, Emilania huxleyi

## Abstract

**Background:**

The advent of Next Generation Sequencing technologies and corresponding bioinformatics tools allows the definition of transcriptomes in non-model organisms. Non-model organisms are of great ecological and biotechnological significance, and consequently the understanding of their unique metabolic pathways is essential. Several methods that integrate *de novo* assembly with genome-based assembly have been proposed. Yet, there are many open challenges in defining genes, particularly where genomes are not available or incomplete. Despite the large numbers of transcriptome assemblies that have been performed, quality control of the transcript building process, particularly on the protein level, is rarely performed if ever. To test and improve the quality of the automated transcriptome reconstruction, we used manually defined and curated genes, several of them experimentally validated.

**Results:**

Several approaches to transcript construction were utilized, based on the available data: a draft genome, high quality RNAseq reads, and ESTs. In order to maximize the contribution of the various data, we integrated methods including *de novo* and genome based assembly, as well as EST clustering. After each step a set of manually curated genes was used for quality assessment of the transcripts. The interplay between the automated pipeline and the quality control indicated which additional processes were required to improve the transcriptome reconstruction. We discovered that *E. huxleyi* has a very high percentage of non-canonical splice junctions, and relatively high rates of intron retention, which caused unique issues with the currently available tools. While individual tools missed genes and artificially joined overlapping transcripts, combining the results of several tools improved the completeness and quality considerably. The final collection, created from the integration of several quality control and improvement rounds, was compared to the manually defined set both on the DNA and protein levels, and resulted in an improvement of 20% versus any of the read-based approaches alone.

**Conclusions:**

To the best of our knowledge, this is the first time that an automated transcript definition is subjected to quality control using manually defined and curated genes and thereafter the process is improved. We recommend using a set of manually curated genes to troubleshoot transcriptome reconstruction.

## Background

Conventionally, genetic and transcriptional studies of non-model organisms have been restricted due to the lack of reference genomes that impede their analyses. Nevertheless, non-model organisms are of great ecological and economic significance; consequently the understanding of their unique metabolic pathways by investigating their gene expression profiles is crucial. The advent of next generation sequencing (NGS) and its continuing improvement, as well as the development of corresponding bioinformatics analysis tools have boosted the number of sequenced transcriptomes in non-model organisms and their automated assemblies have become common over time [1,2].

Numerous software and pipelines have been used to automatically build transcriptomes and several methods that integrate *de novo* assembly together with genome based assembly have been proposed for non-model organisms [3]. Two major alternatives can be employed: 1) Aligning reads to the existing reference genome and then assembling the remaining unmapped reads or 2) Performing a *de novo* assembly first and then using the genome to improve the transcript assembly [3]. However, many open challenges in defining genes remain, particularly where genomes are not available or are incomplete. In spite of the large numbers of transcriptome assemblies that have been performed, quality control of the transcript building process is rarely performed.

Manually defined and curated transcripts or good quality ESTs could be used to assess the quality of automated transcriptome assembly, but to the best of our knowledge, they have not been used. In non-model organisms in particular, it is critical to have genes built from the species being studied, as closely related well-annotated species might not be available. In spite of the great potential importance, the processes used to manually define and curate genes have not been documented until now (Ben-Dor S., in preparation).

In this study, the transcriptome of the bloom-forming alga *Emiliania huxleyi* was built. *E. huxleyi* is a cosmopolitan unicellular photoautotroph that plays a prominent role in the marine carbon cycle [4,5]. Its intricate calcite coccoliths account for a third of the total marine CaCO_3_ production, making it highly susceptible to future ocean acidification [6]. In addition to their role in the biogeochemistry of carbon and related climatic impacts, coccolithophores produce the sulfur containing compound dimethylsulphoniopropionate, precursor of the dimethylsulfide gas which is a major source of sulfur to the atmosphere where it can influence aerosol formation and consequently cloud condensation nuclei [7].

The recently published genome assembly of *E. huxleyi* is a draft that was constructed from Sanger reads [8]. A large number of available unassembled genomic reads, numerous repeats and duplications, as well as holes in the genome, indicated that the genome alone would not provide a good basis for building transcripts. Therefore we opted for an integrative pipeline to build the transcriptome. To test and improve the quality of the automated transcriptome reconstruction, we used 63 manually defined and curated *E. huxleyi* genes, several of them experimentally validated. After each step in the automated definition pipeline, the presence of the manually defined genes was checked, allowing troubleshooting of missing genes and improving our pipeline. This is the first time that an automated transcript definition is subjected to quality control using manually defined and curated genes and thereafter the process is improved.

## Results

### Description of the experimental system

The experimental system was *E. huxleyi* cells subjected to viral infection over a time course, with two different viruses, lytic (EhV201) and non-lytic (EhV163). RNAseq was performed for six samples using the Ilumina HiSeq2000 as follows: control (no virus) and infected with EhV201 or EhV163, at two time points: 1 and 24 hours post infection. The in-depth description of the experiment and its biological significance was submitted elsewhere (Rosenwasser S, Mausz MA, Schatz D, Sheyn U, Weinstock E, Tzfadia O, Ben-Dor S, Feldmesser E, Pohnert G, Vardi A: Rewiring host lipid metabolism is central in infection by large viruses regulating the fate of algal blooms in the ocean, Submitted). Table 1 summarizes the number of reads obtained for each sample after removing adaptors and trimming to 90 bases. The sequences were trimmed because of a decrease in quality scores in late sequencing cycles due to the high GC content (Additional file 1: Figure S1). The sample containing EhV201 for 24 hours had the highest number of reads with adaptor sequences (about 15 million) and a relatively low number of reads, since many of the *E. huxleyi* cells were dead due to the viral infection [9-11] and therefore there was a higher percentage of low quality or degraded RNA (Table 1).

**Table 1:** Read counts

**Sample**	Total reads (cleaned*)	Reads aligned to Emihu1plus**	**Reads mapped to transcripts**
2BE	79,031,642	51,787,959 (66%)	65,282,968 (83%)
4BE	70,232,202	44,631,230 (64%)	57,466,155 (82%)
6BE	71,097,330	44,755,528 (63%)	57,685,982 (81%)
8BE	84,292,692	50,341,359 (60%)	64,839,232 (77%)
10BE	30,393,401	3,559,270 (12%)	6,042,025 (20%)
12BE	74,680,032	44,832,848 (60%)	58,182,177 (78%)

*After removing adaptors and trimming to 90 bp (removed 15million reads in 10BE and up to 5 million in the other samples) **Each sequence was counted once even if several reads have the same sequence, each sequence aligned to the genome ~2-2.5 times in average (for 10BE, 4 times). Percentage of cleaned reads in parentheses.

### Transcriptome assembly

**Figure 1:**
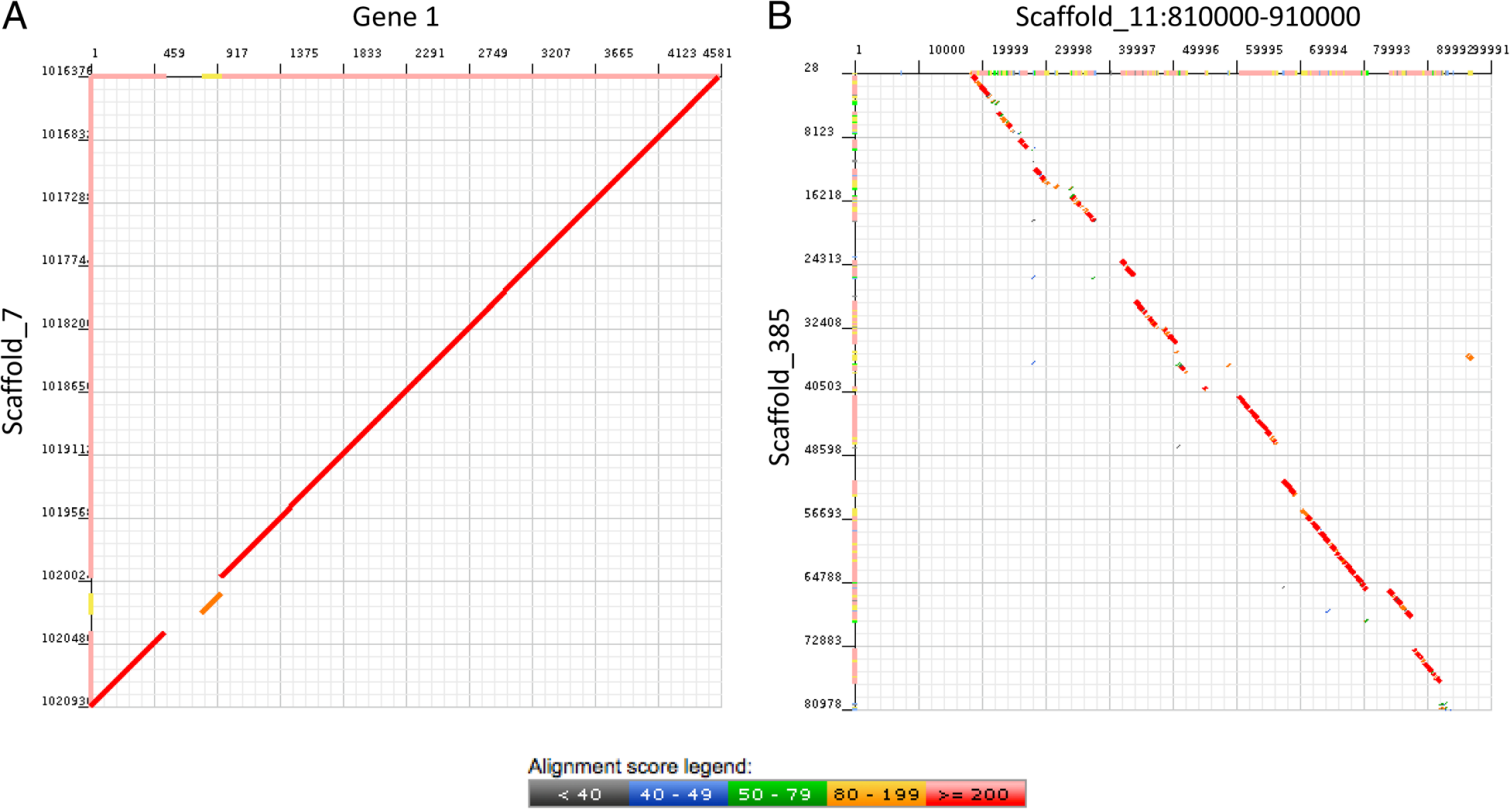
**Genome assembly quality assessment.** Blast alignments were performed at the JGI web site (http://genome.jgi-psf.org/pages/blast.jsf?db=Emihu1) to examine the Emihu1 assembly **(A)** Hole in the genome. “Gold standard” gene 1 was compared to the genome. There is full coverage of the gene in both ESTs and RNAseq reads, but when compared to the genome, there is a part which does not have coverage. **(B)** Genomic Duplication. In building the “gold standard” genes several mapped to two contigs consistently, scaffold_11 from 810,000-910,000 and scaffold_385. Alignment of the two sequences is shown, with the segment from scaffold_11 as the query sequence. Duplication of almost the entire segment can be seen.

**Figure 2:**
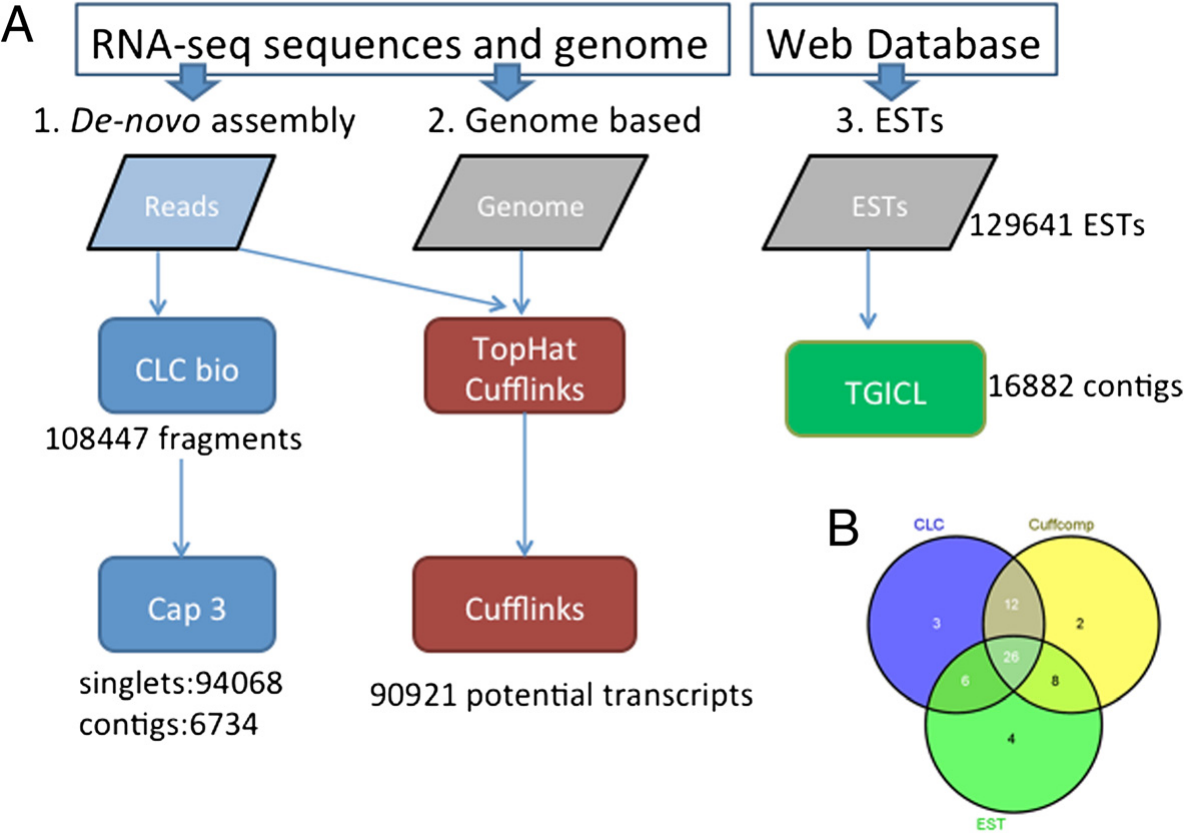
**Initial approaches for automated transcriptome building. (A)** Three different approaches were applied to automatically define the transcriptome, the first one, *de novo* assembly, uses only the reads, the second one uses the reads and an improved version of the genome assembly and the third one is based on a publically available EST collection. See Methods for details. **(B)** Venn Diagram of the “gold standard” genes found in each of the three approaches.

The available genome assembly (Emihu1) is a draft, and was constructed from Sanger reads. There are unassembled reads available, and this together with our experience in hand-curation, where we observed many repeats, duplications, and holes in the genome (Figure 1), led us to believe that additional genomic information is essential to achieve a high quality transcript assembly. In addition to the genome, there were publicly available ESTs, which can provide additional information. In light of this, three different approaches were applied to define *E. huxleyi* transcripts (Figure 2A, Additional file 2), two of them utilizing our RNAseq data. The first was *de novo* assembly; the second was a genome-based alignment to an improved version of the genome assembly. The third approach utilized a collection of publicly available *E. huxleyi* ESTs. All approaches were integrated at the end.

#### *De novo* assembly of transcripts

The adaptor-cleaned and trimmed fastq files were assembled using CLC Assembly Cell. Reads from all samples were pooled for assembly. This resulted in 108477 fragments. In order to reduce redundancy CAP3 [12] was used to cluster the fragments. The outcome was 6734 contigs and 94068 singlets.

#### Genome based definition of transcripts

In addition to the Emihu1 current assembly of the genome, the JGI website database includes Sanger sequencing reads that were not included in the current assembly. They are called unplaced genomic reads and consist of 95120247 bases (106669 N's and 95013578 A, C, G or T) in 161432 sequences. The unplaced reads were assembled using Newbler, resulting in 13227 contigs. To evaluate the possible contribution of these contigs to the transcriptome definition, the reads of Sample 10BE which had the lowest number of *E. huxleyi* reads (Table 1), were aligned to these newly assembled contigs. 742435 reads were mapped to the new contigs, as compared to 3.51 million reads previously aligned to Emihu1. In view of the high number of reads aligned to the new contigs, they were assembled to the available genome using Minimus2 [13] to create an improved genome version, Emihu1plus (Additional file 3).

The reads of each sample were aligned separately to the Emihu1plus genome using TopHat [14]. The total number of reads aligned to the genome per sample spanned from 44 to almost 52 million. The exception was Sample 10BE, as mentioned above, which has only 3.56 million aligned reads due to advanced cell lysis (Table 1). After alignment, Cufflinks and Cuffcompare [15] were applied to all the TopHat outputs to define transcripts. In this process, 90921 potential transcripts were defined.

#### EST collection

ESTs were downloaded from NCBI, using the taxid 2903, for a total of 129641 ESTs. The ESTs were clustered using TGICL. 113756 of them were assembled into 16882 clusters containing at least two ESTs. Single ESTs that were not clustered (15885) were not utilized for further transcriptome reconstruction.

### Manual definition of genes

**Figure 3:**
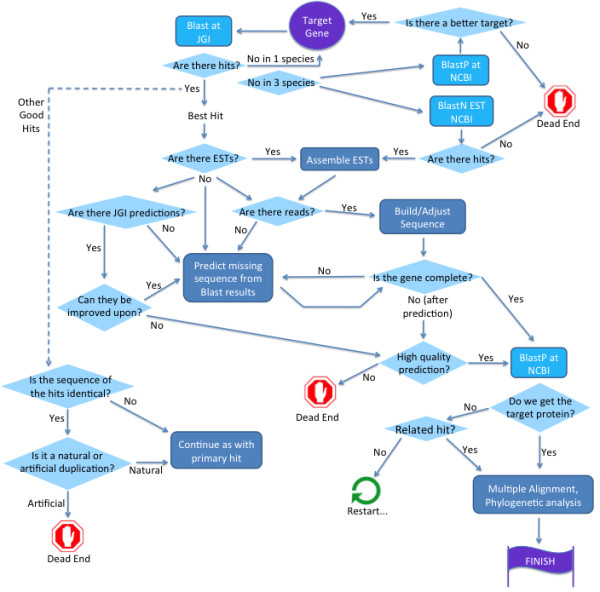
**Manual gene definition procedure.** The procedure of manual gene definition is presented as a decision tree, with the start and end in purple. The procedure starts from the choice of the target gene (purple circle, top middle), which was taken from three species, human, *Arabidopsis thaliana*, and yeast. The chart flows from top to bottom, with decision points in pale blue diamonds, and analyses in blue rectangles (database searches in bright blue, and other analyses in gray-blue).

In parallel to the automated approach for transcript assembly, we manually defined a set of *E. huxleyi* genes. These genes were used in order to assess the quality of the automated pipeline. The manual definition started from the choice of a target gene (Figure 3). Protein sequences of the target gene from human, *Arabidopsis thaliana*, and yeast (*S. cerevisiae*), and if necessary additional species, were compared to the *E. huxleyi* genome on the JGI genome website using TBlastN [16]. Hits were inspected to see if there was any transcript evidence (ESTs). If there were matching ESTs, they were assembled into transcripts, and compared to the predictions, if there were any. If there was incomplete EST coverage, but a JGI predicted gene model, the blast results were used to fix the prediction accordingly. When the RNAseq reads became available, if possible, the putative transcripts were corrected on the basis of the reads. If there was more than one genomic hit in the blast results, each successive hit was checked to see if it was a truly independent hit, representing a family member, or a duplication, which was then classified as real or artificial (Figure 1B). If no ESTs were available to use as an anchor for a predicted transcript, then a combination of reads (if available), prediction based on blast hits and the JGI predictions were used to construct a transcript.

If there was no genomic hit, or if there was a genomic hit with a hole in the middle of a locus (Figure 1A), and in some cases where there were no ESTs in JGI, searches were performed against *E. huxleyi* ESTs in NCBI, in order to identify sequences that might not have been mapped to the genome, generally due to missing sequence in the genomic build. Transcripts were then constructed and extended as far as possible by running Blast.

The sequence was then translated, and a BlastP search was run at NCBI to determine if the putative protein was closest to the target gene in other species, and if it had the proper domains. We found that in many proteins repetitive sequences interrupted the canonical domain composition, and in some cases the domains themselves.

After the protein sequence was finalized, multiple alignments and phylogenetic trees were constructed with protein sequences from representative species to see that the sequence indeed belonged, who its closest relatives were, and in the case of multiple family members, to attempt to assign orthology.

We compiled a set of 63 “gold standard” genes (Sequences in Additional file 4) that were used afterwards for quality control of the automated transcript assembly. Validation of 18 of the sequences was performed by real-time PCR with primers designed to the manually constructed sequences or Western blot (Method of construction and validation status: Additional file 5). All of the sequences but two had reads in the RNAseq data of the current dataset, and the remaining two have reads in an additional dataset (not shown).

The “gold standard” genes all have known functions, come from at least five different biological pathways, and are distributed in various cellular compartments. They include both globular and transmembrane proteins. They are a mix of short and long transcripts ranging from 569 to 4661 base pairs, with varying numbers of exons, ranging from single exon to 17 exon genes, and are expressed in varying levels according to the RNAseq data (Additional file 5). The “gold standard” genes therefore have a wide representation of the various types of genes extant in *E. huxleyi*.

### First round of quality control using manually defined genes

To assess the quality of the transcriptome reconstruction, presence of the 63 “gold standard” genes in the three transcript definition approaches was examined (Figure 2B). The transcripts were compared to the standard genes using Blat [17], with a minimum hit score of 200, in order to ensure significant hits. Four of the genes had less than 10 reads in the RNAseq, and therefore could not be found in the read-based arms of the assembly. In the genome based transcript collection, of the 59 possible genes, eleven were missed. In the *de novo* assembly, twelve genes were missed. In the EST branch, 44 genes were found out of 48 with ESTs. The four that were missed either had only one EST, or non-overlapping ESTs that were therefore not clustered. Two of the four transcripts which did not have enough reads in the RNAseq were found in the EST branch, and two were not found at all.

### Improving the transcript repertoire

**Figure 4:**
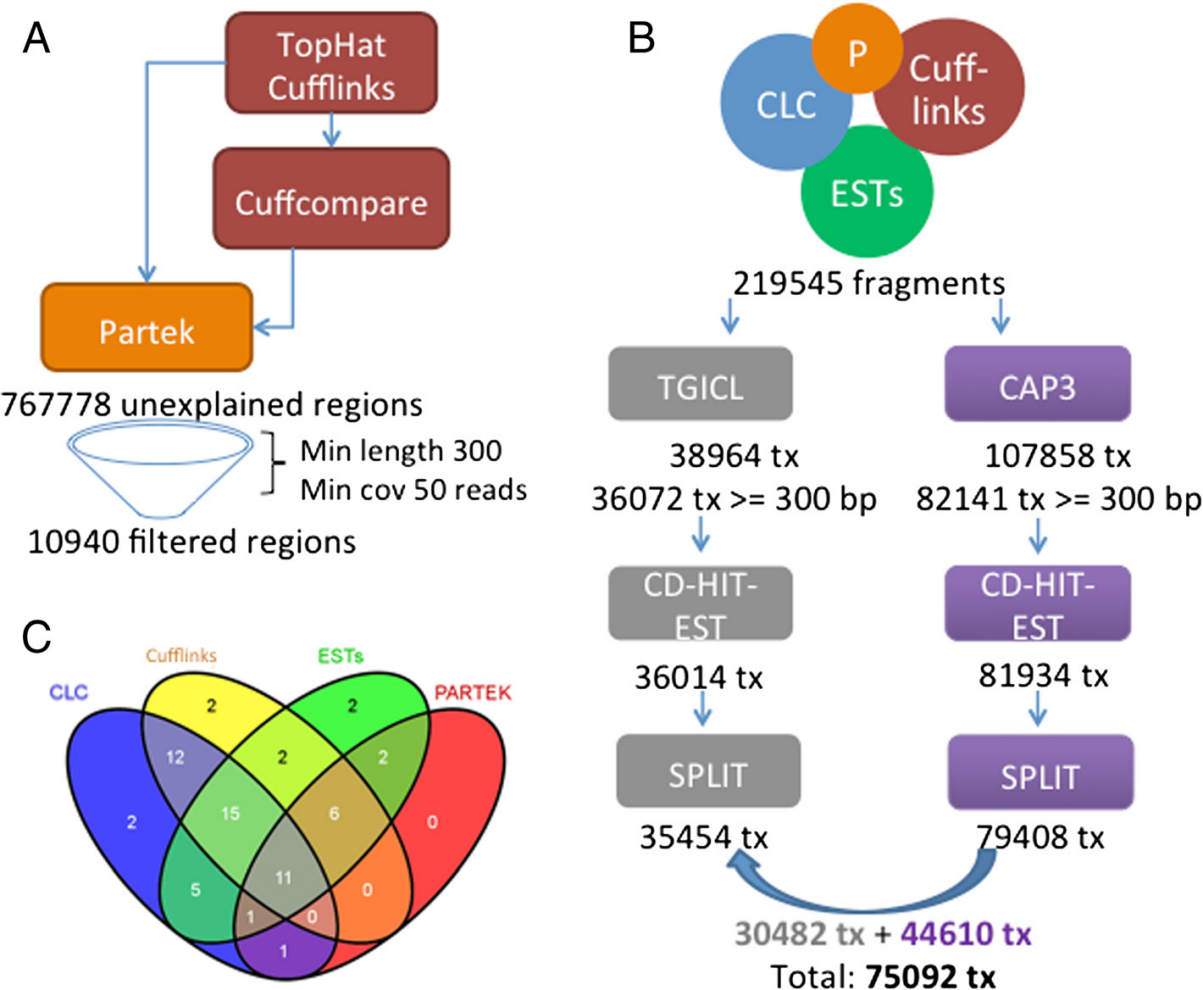
**Automated transcription definition improvement. (A)** Graphical representation of Partek® Genomics Suite™ improvement. Partek found regions that had reads in places that Cufflinks could not define transcripts. Regions with a minimum coverage of 50 reads and a length of at least 300 bp were selected for defining further transcripts. **(B)** Clustering of the transcripts found by the four methods (CLC Assembly Cell, Cufflinks and Cuffcompare, ESTs and Partek) using two different tools (TGICL, CAP3) and combining the results of both. tx = transcripts. **(C)** Venn diagram of the “gold standard” genes found in each of the four approaches.

In order to define the genes missed by the Tuxedo suite [14,15] (TopHat and Cufflinks), the Partek® Genomics Suite™ software was applied. Partek was used to find regions where reads aligned, excluding regions with Cufflinks transcripts (Figure 4A). These regions were required to have a minimum length of 300 base pairs and a minimum coverage of 50 reads, as the default Partek settings are permissive. Of the 767778 “unexplained” regions defined by Partek, 10940 met the requirements (Figure 4A).

In comparison to our “gold standard” collection, Partek found only twenty-one genes, but that was due to the exclusion of previously defined transcripts. Of the twenty-one, it found four that were missed by Cufflinks, two of which had also been missed by CLC Assembly Cell. The other 17 genes that were found by Partek added new fragments to transcripts defined with Cufflinks. All told, using all four approaches, 61 out of the 63 “gold standard” genes were found (Figure 4C).

For each of the approaches, we characterized the transcripts and their translations. The median transcript length for the *de novo* fragments was 303 bp, with a median ORF length of 98 amino acids, and a median ORF coverage of 99%. The other methods resulted in longer sequences (median range 624–892 bp, 170–259 amino acids), but a lower ORF percent coverage, 89% for all arms (Additional file 2).

The transcripts defined by the three primary approaches and by Partek, 219545 sequences, were clustered using TGICL (Figure 4B, Additional file 2). This process resulted in 36072 transcripts longer than 300 base pairs, including contigs and singletons as defined by TGICL. To further remove redundancy, CD-HIT-EST [18] was applied and the number of transcripts was slightly reduced to 36014.

The next round of quality control was performed on the set of 36014 transcripts. Only 53 of the “gold standard” set were found, indicating that in our clustering 8 genes were lost. Inspection of the output of both programs showed that the genes were lost by TGICL.

### Quality control on the protein level

The next step was to check for the presence of the “gold standard” genes on the protein level. The transcripts were translated taking only the longest open reading frame (ORF), with the ORF defined from stop to stop. We did not require an initial Methionine, as many of the transcripts are incomplete. Only 36 (68%) of the standard set were found on the protein level. Missed proteins were inspected on a case-by-case basis, to understand how they were lost and improve the pipeline.

**Figure 5:**
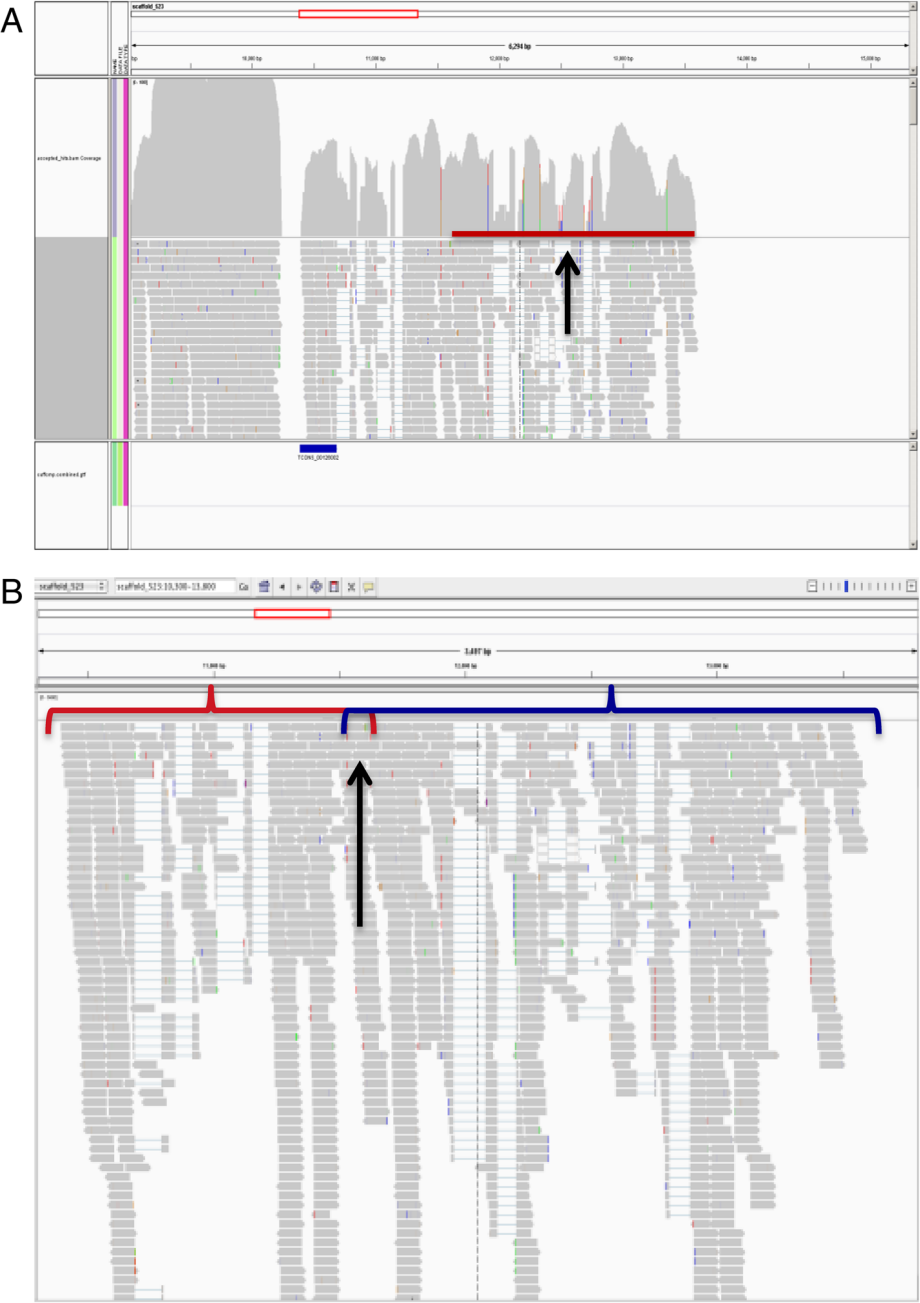
**Problems in the automated transcriptome reconstruction. (A)** Transcripts missed, even with full coverage. The red line (pointed out by black arrow) gives an example of a gene with a large amount of reads that was not defined by Cufflinks. Upper part of the plot shows the read coverage, middle part the reads and lower part the transcripts defined by Cufflinks as blue bars. **(B)** Overlapping transcripts. An example is given of two transcripts overlapping in their 3’ UTRs (one manually and the other Cufflinks defined). The transcripts are represented by red and blue lines and their overlap by a black arrow. Visualization of reads on genome in both panels performed using IGV browser [19] (http://www.broadinstitute.org/igv/).

Three causes were identified: 1) Frameshifts due to either sequence or assembly errors; 2) Correct reading frames were shorter than the longest incorrect frame (Additional file 6); 3) Fusion of transcripts on opposite strands due to overlaps in 3’ UTRs (Figure 5). In order to improve the fusion transcripts, we decided to split them. For each transcript the three longest ORFs were examined, and in cases where translations derived from different ORFs did not overlap and the resulting peptides were at least 100 amino acids length, the transcripts were split.

The process ended up with 1559 sequences split into two and 50 into three potential transcripts. 759 of the split transcripts were found to be transcribed from opposite strands. Redundancy of the whole set after splitting was removed at the protein level using CD-HIT [13], resulting in 35454 transcripts. When this set of translations was compared to the 53 transcripts of the standard set that were detected, 42 proteins out of the 53 were found (79%) an improvement of 11%.

### Additional improvement of the transcriptome

At this point, it was clear that TGICL lost transcripts during the clustering process. To overcome the problem of missing genes, the initial transcripts from the four approaches were clustered using CAP3 (Figure 4). Further redundancy was removed by CD-HIT-EST. The number of potential transcripts longer than 300 base pairs was 79939. In order to split artificially fused transcripts, the algorithm developed to split the sequences previously was used. 2393 sequences were split in two, and 66 were split in three. Once again, approximately half of the split sequences (1143) were from opposite strands. Redundancy at the protein level was removed with CD-HIT, and subsequently the redundant transcripts were removed, resulting in 79408 defined transcripts. These transcripts were compared to the standard set, and 61 out of 63 transcripts were found.

Two different clustering algorithms were applied to the collection of potential transcripts. TGICL strongly removed redundancy, and makes longer transcripts (median 1242 bp) but loses genes. CAP3 does not lose genes, but makes shorter transcripts (median 907 bp) and leaves redundancy in the collection (Additional file 2). To take advantage of both algorithms, the two collections were compared. Transcripts defined by CAP3 that were identical (19845) or were contained in (14953) TGICL defined transcripts were replaced by their matching counterparts in TGICL (30482). This reduced the number of transcripts by 4316. Additional transcripts defined by CAP3 that did not have a good match to TGICL defined ones (44610) were introduced into the final collection without changes. The final collection included 75092 transcripts.

The final collection was compared to the “gold standard” set both on the DNA and protein level. 61 transcripts and 47 proteins were found, an improvement versus the TGICL alone. The transcripts that were not detected on the protein level were analyzed in depth to discover the possible flaws in our pipeline. There were two major causes, one on the transcript level, and one in the translation process. On the transcript level, we discovered that many of the genes had both spliced forms and intronic read-throughs (intron retention) (Additional file 1: Figure S9, Additional file 6, Example 1). The retained introns disrupted the coding sequence. The second major cause of missing protein sequences was due to our definition of an open reading frame from stop-to-stop (Additional file 6, Example 2).

We examined whether 63 genes were enough for quality control by increasing the number of hand constructed genes to 100, and running comparisons on the DNA and protein levels at all stages of transcriptome reconstruction. The results show no change in the percentage of genes or proteins found in any of the steps (Table 2). The additional 3 transcripts and 9 proteins that were not found at the final stage were due to reasons seen previously, lack of reads or longer irrelevant open reading frames, respectively.

**Table 2:** Evaluation of enlarged “gold standard” gene set

	**Transcripts**		**Peptides**	
	**63**	**100**	**63**	**100**
De Novo (CLC assembly cell)	75%	72%	-	-
Cufflinks	76%	78%	-	-
Partek genomics suite*	33%	24%	-	-
ESTs	70%	69%	-	-
TGICL	84%	81%	66%	62%
CAP3	97%	95%	75%	75%
Final collection	97%	95%	75%	75%

The percentage of sequences found by each step of the transcriptome reconstruction is given.

*Partek percentages are lower as the program was used to define transcripts in very limited regions.

### Characterization of the E. huxleyi transcriptome

**Figure 6:**
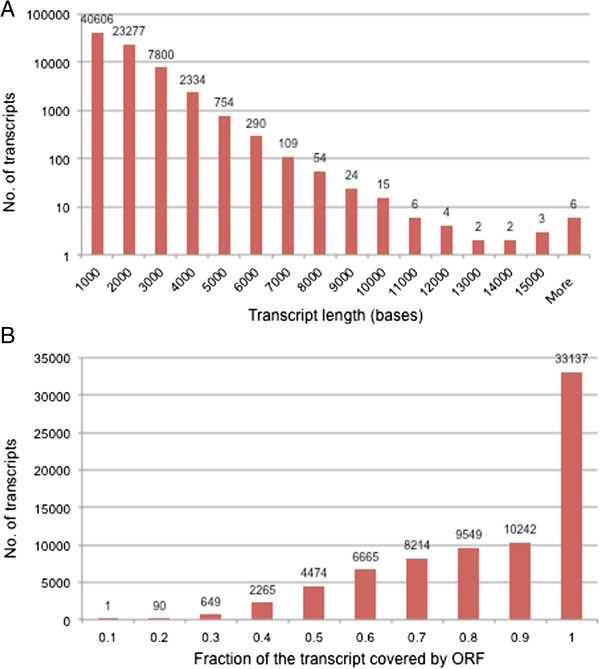
**Automatically defined transcriptome characteristics. (A)** Transcript length frequency. **(B)** Fraction of transcript covered by ORF. Numbers in the top of the bars represent the number of transcripts in each length or fraction bin.

The 75092 transcripts range in length from 301 to 34193 base pairs (bp). Close to half of the transcripts (34680) are more than 1000 bp long, with an additional 32% (23993) longer than 500 bp (Figure 6A). 70% of the transcripts had ORFs over more than 80% of their length, and 44% had ORFs covering the entire length of the transcript (Figure 6B).

In order to assess how well the reads fit the final transcripts, the reads were mapped to the transcript collection, and 80% of the total reads mapped fully (Table 1), in all samples except for sample 10BE that included a high percentage of viral reads.

The final transcript collection was compared to the JGI ‘Best transcript’ models (39126 models) [8] by applying Blat. Our collection was used to query the target models. Out of 75092 transcripts, 63% (47358) were found to have at least a partial match to the predictions, and only 7.4% (5578) of the transcripts matched in at least 90% of their sequences (Additional file 1: Figure S2). Of the 39126 models, 87.5% (34248) had at least partial coverage of our transcripts. We compared the InterPro annotations of the JGI matching and non-matching transcripts, and while only 29% of the JGI matching transcripts had annotation, 45% of the non-JGI transcripts did.

**Figure 7:**
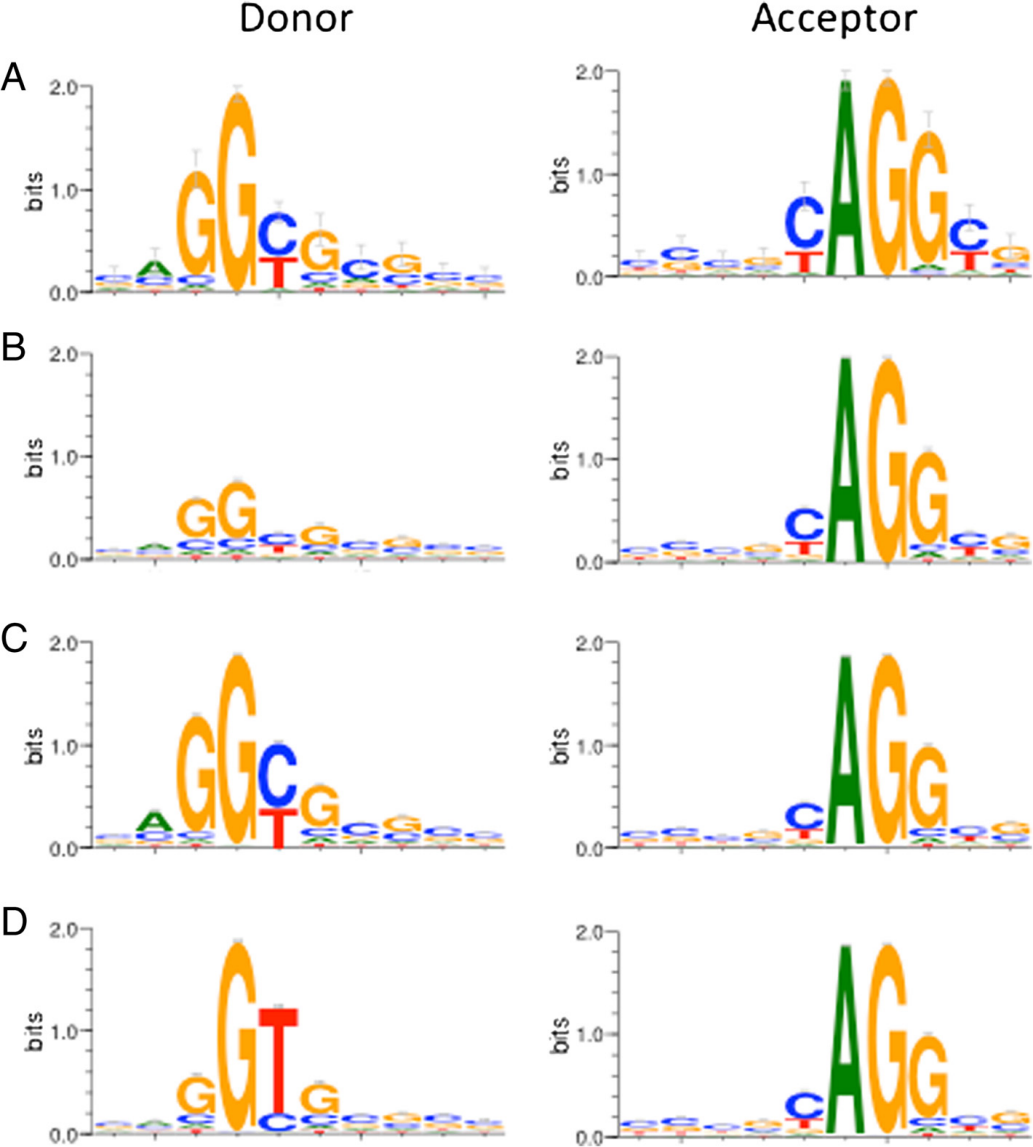
**Splice junctions in *E. huxelyi*.** Splice junction donor and acceptor sites logos in **(A)** “Gold standard” manually defined genes **(B)** NCBI ESTs **C)** Cufflinks defined transcripts and **(D)** JGI ‘Best transcript’ models gene predictions. Logos constructed with WebLogo 3.3.

During the manual definition of the genes, 241 splice junctions were mapped to the genome sequence, and unlike published genomes, a prevalence (56%) of non-canonical GC-AG junctions was observed (Table 3, Figure 7A). The GC junction was used only when it was obligatory, and there was no other way to map the sequence (particularly for those based on ESTs, 48 out of 63 transcripts). Thirteen of these junctions (9 GC, 3 GT and 1 GA) were verified with reverse transcriptase PCR and Sanger sequencing (Table 4, Additional file 1: Figure S8).

**Table 3:** Splice junction sequence distribution

**Donor**	**GC**	**GT**	**GA**	**AT**	**CT**	**Others**
Gold standard	135 (56%)	95 (40%)	11 (4%)			
ESTs	13266 (39%)	4708 (14%)	1203 (4%)	414 (1%)	1122 (3%)	13376 (39%)
Cufflinks	44991 (61%)	27374 (37%)		708 (1%)	285	
JGI best transcript	80404 (82%)	14387 (15%)	1180 (1%)	108	427	1135 (1%)
**Acceptor**	**AG**	**AC**	**GC**	**AT**		**Others**
Gold standard	241 (100%)					
Cufflinks	70512 (98.6%)	812 (1%)	182	29		
JGI best transcript	95694 (98%)	250	277	75		1331 (1.4%)

**Table 4:** Manual validation of splice donor sites

**Donor**	**GC**	**GT**	**GA**	**Others**
RNA-seq reads	175	94	1	
Sanger sequencing	9	3	1	
ESTs	167	94	6	2

Further investigation of this finding was performed with additional junction collections, based on ESTs or RNAseq reads, with both automated and manual analysis. The full collection of *E. huxleyi* ESTs was mapped to the genome using Blat [13]. The mapping results were very noisy, so for automated analysis the donor junctions were filtered for AG acceptors (34089 final junctions). The GC to GT ratio was approximately 3:1, further corroborating our initial finding (Table 3, Figure 7B). In addition, 269 junctions from 32 EST-based transcripts in 25 randomly chosen genomic loci were manually aligned and examined, and the percentage of GC junctions was 64% (Table 4).

Splice junctions from automatically generated Cufflinks transcripts were also analyzed, and donor (73358) and acceptor (71535) sites were extracted from Emihu1plus. In order not to count the same junction twice in cases of alternate splicing, non-redundancy was performed on the genomic coordinates. The most prevalent splice donor is GC (61%, Table 3, Figure 7C) while the canonical sequence GT was only used 37% of the time. The splice acceptor is almost entirely the canonical AG (98.5%). The even less canonical sequences (for example a donor not starting with a G), or at least part of them, represent misalignments of TopHat (Additional file 1: Figure S3). Validation was performed by manual inspection of spliced RNASeq reads mapped to the genome, representing 36 randomly chosen transcripts. A total of 270 junctions were inspected, and GC was found in 65% of the donors, while GT was found in 35% (Table 4, Additional file 1: Figure S9). In contrast, the JGI ‘Best transcript’ models have the opposite distribution, with 82% GT and only 15% GC (Table 3, Figure 7D).

Intron retention was observed as a major cause of redundancy in the transcript collection and loss of proteins in the translations. Therefore the phenomenon was assessed in an attempt to quantify the level of intronic reads and additional types of alternative splicing. Currently available programs to determine types of splicing could not be used, for two reasons. Firstly, many have a transcript mapping step which relies on GT-AG junctions, which is inappropriate for *E. huxleyi*. Others require a database of defined splice junctions, which is problematic to create due to the pervasive nature of the intronic reads (Additional file 1: Figure S9). A manual inspection was performed on 535 introns taken from 68 loci, and 89% (478) showed reads throughout the length of the intron. The other instances of alternative splicing observed were: 8 exon skipping, 6 alternate acceptors, and 5 alternate donors.

### Transcriptome annotations

The final transcripts were annotated using Gene Ontology (GO) [20] terms by applying Blast2GO [21] at the DNA level. 61% (45704) of the transcripts had at least one Blast hit. GO terms were transferred to 84% (38510) of the transcripts with Blast hits, and finally, GO terms were successfully assigned to 40% of the total transcriptome (29868), after setting the Annotation Cut-Off to 45 (Additional file 1: Figure S4). The total number of annotations was 143794; the mean number of GO annotations per gene was 5 and the mean level in GO was 5.5 (Additional file 1: Figure S5). The most frequent organism in the Blast top hits was *Afipia broomeae* with 7071 hits and the second one was *Aureococcus anophagefferens* with 2650 (Additional file 1: Figure S6). *A. Broomeae* is a very well annotated species, and that is probably the reason for the large number of hits, as it is phylogenetically distant from *E. huxleyi*. The other species are all relatively closer. The top GO terms to which transcripts were assigned in the categories: biological process, molecular function and cellular component were oxidation-reduction process, ATP binding and integral to membrane respectively (Additional file 1: Figure S7). A list of terms and the number of genes annotated to them can be found in Additional file 7.

KOG and KEGG annotations were performed at the protein level; 2675 proteins were annotated in the KOG system and 5850 in KEGG (Additional file 7).

## Discussion

Definition of transcripts can and needs to be improved from the current standards. Fully automated transcript building, while the only practical method on a large scale, can be improved, particularly for the individual genome. We recommend a manually curated reference set for quality control per genome, which will allow detection of genome specific issues, and allow improvement of existing tools and pipelines. Once done, the new pipeline can then be utilized for all future transcriptomes of the same genome. In our particular case, *E. huxleyi*, the genome has non-canonical splice junctions, overlapping genes, duplications and holes in the genome. These issues were addressed, to the best level possible, utilizing the “gold standard” set of genes for quality control. We have since successfully analyzed additional transcriptomes from the same organism, taking these issues into account.

### Assembly strategy

One of the goals of this study was to achieve a complete picture of the transcriptome: utilizing all the available data, build accurate transcripts without either missing genes, or including spurious sequences. The data at hand was the genome assembly from JGI, ESTs from GenBank, and our RNAseq reads. The genome was incomplete, and the EST coverage was very partial, with only 129,000 sequences for a genome with an estimated 30,000 genes, and many more transcripts. The RNAseq data had good read depth, however, reads were relatively short (90 bp) and transcript coverage was incomplete due to the low complexity and highly repetitive nature of the underlying sequence.

We used three different approaches to assemble the sequence: two utilized the reads, one genome based and one *de novo*, and an EST only branch. For the read-based arms, the first decision that had to be made was whether to combine the reads from the different biological samples or to analyze them independently. For the genome-based approach, we chose to analyze each lane individually (Cufflinks), and then join them (Cuffcompare), while for the *de novo* assembly (CLC Assembly Cell), without the basis of the genome to build on, we used all the lanes together in order to maximize the chances of building a transcript, particularly those with low expression levels. The next decision was whether or not to include the ESTs in the *de novo* construction, as ESTs allow elongation of putative transcripts due to their length and different sequencing method (Sanger). We decided to cluster and analyze the ESTs on their own first, in order to improve the ESTs’ reliability, and to combine the various tracks afterwards.

When the analysis was performed, the programs chosen were considered state-of-the-art. New tools have been developed in the interim, such as Trinity [22] and SOAPdenovo-trans [23] among others. Programs developed for genome assembly and later adapted to transcript assembly (SOAPdenovo-trans, Oases [24]) seem to be less accurate than purpose-built transcriptome tools (such as Cufflinks and Trinity, personal communication). While some of the newly developed tools may perform better building transcripts, a manually curated gene set is still highly recommended for troubleshooting, particularly for non-model organisms.

### Manually constructed and curated transcripts

The process of manual construction of genes is highly complex. Complications arise in cases when the target sequence is not related closely enough to the genome of interest to rely on as a basis for comparison, and when there are no cDNA or genomic sequences to compare to. We have both obstacles in *E. huxleyi*, but the first is the greater issue, as there are no close relatives, and each gene has a unique history, some closer to plants, some to mammals, some even to bacteria (Additional file 1: Figure S6).

Various techniques are used to bridge the gaps, many of which can be implemented manually, but are difficult to automate, particularly integration of information from multiple sources. This is particularly true of the human ability to process data based on visual presentation. Theoretically, any one of these techniques may potentially be programmed, but in practice, it is not trivial, and the combination of methods for the decision-making process is not possible in the computer capabilities currently available.

### Quality control

One of the major goals of this project was to test the quality at each step of the transcriptome construction. In the very first step, examination of the reads, we found that the reads had to be trimmed due to a noticeable drop in quality towards the end (last 10 cycles, Additional file 1: Figure S1) due to the high GC content.

The manually curated genes gave us the opportunity to detect faults in the process of automated transcript building. When we compared the “gold standard” to our initial gene build, many transcripts were missing. The loss of genes was caused by: 1) Genes that were only predicted, with no transcript evidence at all; 2) Genes that had coverage, but by only one EST and had no reads; 3) Genes with no reads and more than one non-overlapping EST; 4) Genes without enough sequence coverage for an automated program to build a long enough transcript; 5) Genes that had read coverage, but Cufflinks and CLC Assembly Cell did not build. We improved our initial build by using the Partek Genomics Suite to define transcripts missed by the earlier methods, and indeed, found all the missing genes that had coverage on the nucleotide level.

### Lost in translation

After we found all the “gold standard” transcripts possible in the dataset, we compared the hand curated and automated datasets on the protein level. Many genes were lost on the protein level, even though they could be detected on the DNA level. The first class of missing proteins was due to artificial joining of transcripts. This phenomenon has been observed in model as well as non-model plants [1]. Transcripts were joined in several ways including on opposite strands overlapping in the 3’ UTR, and on the same strand with some overlapping reads (possibly due to the repetitive nature of the genome). It is interesting to note that we did not observe any overlapping transcripts in the coding regions. We corrected many cases by using a script to split transcripts, based on non-overlapping open reading frames.

The second class of missed proteins was due to the choice of the longest open reading frame, from stop to stop. In some cases, full ORFs from Methionine to stop were in the correct frame and longer than stop to stop (examples in Additional file 6), and in other cases, the functional protein was not the longest frame. We decided to use stop to stop because many of the transcripts were partial, and did not expect to see a methionine in these cases. Another reason was that frame-shifts may cause the methionine to be lost. It may be worthwhile to build a program that will look at both stop to stop and Met to stop and choose the longer of the two. For the cases where the longest frame was not the functional one, an additional check can be performed, for example utilizing BlastX results to choose the proper frame. These issues are compounded in our particular case due to high percentages of intron retention and genomic repeats.

### Additional observations

An additional observation that resulted from the hand curation is the high percentage of non-canonical splice junctions. In the hand curated transcripts, ESTs, and Cufflinks defined transcripts, there is a clear majority of GC splice donors (~60/40 GC/GT). In contrast, the JGI “Best transcript” collection has the opposite ratio (~20/80 GC/GT). The probable cause of the shift from GT to GC is the high GC content of the *E. huxleyi* genome, 65% [8], among the highest of all eukaryotes sequenced to date. This can create a burden on the splicing machinery, as the canonical junctions are 50% AT (GT-AG). The splice donor GC is also seen in many other species, though at very low percentages, but the ‘universal’ splice machinery knows how to deal with GC, which may be why the splice acceptor remains completely canonical. The “new” canon for *E. huxleyi* GC-AG, is 25% AT, and is much closer to the DNA at hand. The JGI “Best transcript” collection is composed of 68% *ab initio* predicted genes [8]. Most of the *ab initio* algorithms are programed to look for canonical GT-AG junctions, and will only use non-canonical if there is nothing else. This leads to the many mispredicted junctions in that dataset.

The non-canonical splicing may contribute to the high frequency of intron retention. This phenomenon was observed both in the manually curated as well as automatically processed transcripts. From the hundreds of alternate events inspected manually, mainly intron retention was observed. It is not clear if the transcripts with intron retention are functional, as in virtually all cases checked, it led to premature stop codons in the putative protein and disturbed the domain structure of the potential proteins (data not shown). This raises the following questions: 1) Is the nonsense-mediated decay mechanism functional in *E. huxleyi*? 2) To what extent is the splicing machinery conserved? 3) Is the efficiency of splicing affected by the change in the junction sequence? Another possible explanation for the observed intronic read-throughs is the presence of unspliced pre-mRNA in the RNA used for sequencing. We view this as unlikely, as the RNA was polyA selected, though it cannot be ruled out, as this may also be different in *E. huxleyi*. These issues will have to be resolved by further research into the various mechanisms mentioned.

### Comparison of programs

Cufflinks missed putative exons and transcripts, where Partek Genomics Suite was able to define them. Partek describes reads mapped to the genome, while Cufflinks attempts to build gene structure. There was no obvious reason for the transcripts to be missed by Cufflinks, as these regions had a minimum of 50 reads and length of 300 bp. It may be that the high rate of intron retention did not allow Cufflinks to predict transcripts in those regions.

We clustered the transcripts using both TGICL and Cap3. While TGICL utilizes Cap3, due to its initial Blast stage, it gives very different results. While TGICL reduced redundancy very efficiently, it lost many transcripts. On the other hand, Cap3 alone clustered well, but left redundancy in the dataset. In both cases, it is not clear why the programs did not perform optimally. As there is no explanation of how or why sequences are removed by TGICL, we were surprised by this finding. Cap3 left the redundancy in the dataset due to the many alternative isoforms.

The final dataset has redundancy, mainly due to putative splice variants. Due to the high level of intron retention, many transcripts are predicted both with and without introns. The programs cannot differentiate between real splicing and spurious splicing, though one possible way to discern correct transcripts would be to take intact reading frames into account.

### Parameters to measure quality of the transcriptome

Using the manually curated transcripts, we found all of the transcripts possible given the constraints of minimum coverage per transcript. On the protein level, we found most of the genes, but have room to improve the pipeline. In independent measures of quality, the length of the transcripts was reasonable (median length of ~1000 bp), and 70% of the transcripts had ORFs over more than 80% of their length. Most reads and ESTs mapped to the final transcripts. Taken together, it indicates a good quality build.

We compared our set of transcripts to the JGI ‘Best transcript’ models, which are mostly based on gene prediction. While most of the JGI set was found in our transcript collection, we had many transcripts not covered at all by the JGI set (37%). This raises the question of fairness of comparison: Firstly of a predicted versus a sequence based set, and secondly our full transcriptome with isoforms included to the ‘Best transcript’ collection. While the JGI models were partially sequence based, and many of the predictions had some evidence of existence in their RNA tag sequencing, the incompleteness of the genome caused issues for proper gene prediction [8]. The predictions suffered additionally due to the fact that they relied on canonical splice junctions, while in *E. huxleyi*, the majority of junctions are non-canonical. While the ‘Best transcript’ models generally have one isoform per locus, the full transcript set has additional transcripts of previously covered loci, while we have coverage of many loci not covered by JGI at all.

While this manuscript was in preparation, a study on the sulfate deficiency response in *E. huxleyi* was published [25], where RNAseq was also performed. They found, as we did, that the JGI models were severely lacking, and do not give good coverage of the transcriptome. We cannot compare our results to theirs, as their sequences are not yet available in a public repository.

## Conclusion

As has been suggested [3] a reference set of transcripts should be generated for quality control. We have shown that the reference set should be genome specific and manually defined, and that it is critical for troubleshooting and accurate quality control. This is the first time, to the best of our knowledge, that a manually built set of genes has been used to improve a transcriptome pipeline. The advantages are clear; it gives an organism-specific picture of the pitfalls and problems that accompany any *de novo* transcriptome sequencing, particularly for non-model organisms.

## Methods

### Improved assembly of the Emihu1 genome

The current assembly of the *E. huxleyi* genome: Emiliania_huxleyi_CCMP1516v1.0 (Emihu1) was downloaded from the JGI (Joint Genome Institute) website. In addition, reads that were not introduced in the assembly were obtained using the command wget http://genome.jgi-psf.org/Emihu1/download/Emihu1_unplaced_genomic_reads.fasta.gz. The unplaced reads were assembled using the Newbler runAssembly program, version 2.3 (http://my454.com/products/analysis-software/index.asp) using default parameters. Newbler was chosen as it is the standard tool for 454 reads and therefore it also works for longer sequences like Sanger reads (which were used in the genome build). The resulting contigs were merged to the Emihu1 genome utilizing the Minimus2 software from the Amos package, version 3.0.0 (http://sourceforge.net/apps/mediawiki/amos/index.php?title=Minimus2) [13].

### Transcriptome assembly and definition

Sequence reads were deposited in GenBank SRA with the study accession number SRP017794.

Sequence reads were trimmed to a length of 90 bp, and adaptors were removed using the cutadapt program [26].

*De novo* assembly was performed with the CLC Assembly Cell (EMEA, Aarhus N, Denmark, http://www.clcbio.com/products/clc-assembly-cell/) software, version 3.2.2. All the lanes were run together using default parameters. The genome based assembly was performed using the TopHat software, version 1.3.0 [14], and was run for each sample separately using the Emihu1plus as the reference sequence and set to the default parameters, except for the minimum intron length that was set to a minimum of 30. Then Cufflinks (version 1.1.0) was run for each sample using the accepted_hits.bam file as input and the -u --min-intron-length 30 options. Cuffcompare [15] was applied on the Cufflinks output of all the lanes together to define a list of transcripts that are comparable between all the samples. Transcript sequences were extracted using the Cuffcompare gtf file coordinates utilizing the Galaxy tool: Extract Genomic DNA (https://main.g2.bx.psu.edu/). Additional partial exons or transcripts missed by Cufflinks were extracted from the alignments using Partek® Genomics Suite™ software, version 6.5, Partek Inc., St. Louis, MO, USA. EST clustering was performed using TGICL version 2.1 (http://compbio.dfci.harvard.edu/tgi/software/). Additional clustering of potential transcripts was performed with TGICL, CD-HIT-EST (version 4.5.4) [18] or CAP3 (Version date: 10/15/07) [12].

ORF extraction and transcript splitting were performed using in-house Perl scripts (available on request). The rules used for splitting were as follows: 1. Transcripts whose ORF covered at least 80% of the sequence were not split 2. Transcripts shorter than 2000 bp were not split, 3. Transcripts whose ORF covered between 10% and 80% of their sequence were split into 2 or 3 if the new ORFs did not overlap and the new transcript was at least 300 bp long. Transcripts whose ORF covered less than 10% of the sequence were removed from the data set. All sequence comparisons carried out for quality control or for any other purpose were performed by with Blat, version 34 × 12 [17].

### Manual definition of genes

The RefSeq (NCBI, http://www.ncbi.nlm.nih.gov/, Gene database) protein sequence of the target genes were taken from *Homo Sapiens*, *Arabidopsis thaliana* and *Saccharomyces cerevisiae* sequentially, starting from human, unless we had reason to believe that a different species might have a better chance of hits. The initial search was TBlastN [16] at JGI (http://genome.jgi-psf.org/pages/blast.jsf?db=Emihu1) with the following parameters changed: Target database: Emiliania huxleyi v1 scaffolds (unmasked), ‘Filter low complexity regions’ off, and ‘Perform gapped alignment’ on.

The hits from the various input species were compared to see if they hit the same genomic loci. Each locus was then inspected, starting with the hit with either the highest score or the longest region of similarity, to see if any transcript evidence was available – ESTs, as seen in the JGI browser, and after we sequenced the transcriptome, the reads mapped to IGV. If there were ESTs available, they were assembled into transcripts using Sequencher version 5.0 (GeneCodes Corporation, Ann Arbor Michigan), and compared to the predictions available at JGI (if there were any). If there was incomplete coverage, the putative transcript was improved using the JGI gene models and the Blast results. When the reads became available, the transcripts were corrected on the basis of the reads. If more than one genomic locus was found in the initial Blast, each successive hit was examined to see if it was truly an independent hit, representing a family member, or an artificial duplication, where the genomic or EST sequences was identical to a previously examined locus.

If no ESTs (or reads) were available to use as an anchor for a predicted transcript, then a combination of the Blast hits and available predictions were used to construct a transcript.

If there was no genomic hit, or if there was a genomic hit with a hole in the middle, TBlastN searches were performed at NCBI (http://blast.ncbi.nlm.nih.gov/), database ‘Expressed sequence tags’, limited to organism taxid: 2903. Transcripts were then constructed and extended as far as possible by running sequential BlastN searches.

If there was no hit either in JGI or in NCBI, with the proteins from the three initial species, a BlastP of the initial protein sequences was performed at NCBI, looking for hits in other algae or related marine species, and bacteria as well. These sequences were then used as input to the Blast searches as described above.

The putative transcript sequences were compared to the reads mapped to the genome, visualized with IGV version 2.0 (http://www.broadinstitute.org/igv/), and improved where possible. The sequences were translated using NCBI ORF Finder (http://www.ncbi.nlm.nih.gov/gorf/gorf.html) [27] for full reading frames, and Expasy Translate (http://web.expasy.org/translate/) for incomplete reading frames. BlastP was performed at NCBI to determine if the protein matched the target gene, and if it had the proper domains. If domains weren’t found, domain searches were performed using InterProScan (http://www.ebi.ac.uk/Tools/pfa/iprscan/) [28], Pfam (http://www.pfam.org) [29] and Prosite [30] (http://prosite.expasy.org/).

The final protein sequences were aligned (using ClustalW version 2.1, and Muscle version 3.8.31) [21,31] to the initial target proteins, the closest blast hits, and other species as necessary, and phylogenetic trees (ClustalW version 2.1, Neighbor Joining with 1000 bootstraps, and Phylip version 3.69 Proml with 100 datasets, 3 repeats, 9 jumbles) [21,32] were constructed and orthology assigned where possible.

### Characterization of transcriptome

Splicing junction sequences were extracted based on gtf files of the transcripts (output of Cuffcompare, or downloaded from JGI) as compared to the relevant genome (Emihu1plus, Emihu1 respectively). The EST sequences were mapped to the genome using Blat. A gtf file was extracted from the alignments using the script http://code.google.com/p/popgentools/source/browse/trunk/misc/psl2gtf.pl?spec=svn2&r=2. Donors with non-AG acceptors were filtered out for the ESTs. The strand was taken into account, and redundancy was removed to leave a single copy of each junction. The sequences were extracted using Galaxy ‘Extract flanking sequences’. Logos were constructed using WebLogo version 3.3 (http://weblogo.threeplusone.com/). Venn diagrams were constructed using Venny (Oliveros, J.C. (2007) VENNY. An interactive tool for comparing lists with Venn Diagrams. http://bioinfogp.cnb.csic.es/tools/venny/index.html).

### Transcriptome annotation

The full collection of transcripts was annotated at the DNA level using Blast2GO (http://www.blast2go.com) [33], the ‘Annotation Cut-Off’ was set to 45. This Annotation Cut-Off represents the maximum similarity weighted by GO evidence codes (http://www.blast2go.com/data/blast2go/b2g_user_manual_13012013.pdf). KEGG and KOG annotations were obtained by submitting the longest ORF protein sequence for each transcript to the WebMGA server [34] (http://weizhong-lab.ucsd.edu/metagenomic-analysis/) and the outputs downloaded.

## Abbreviations

NGS: Next generation sequencing; EST: Expressed sequence tag; JGI: Joint Genome Institute; GO: Gene ontology.

## Competing interests

The authors declare that they have no competing interests.

## Authors’ contributions

EF, SR, AV and SBD planned the study. EF and SBD designed, implemented, and performed the analysis. SR and AV performed the biological experiment and provided the data. EF and SBD wrote the manuscript, SR and AV contributed to the manuscript, and all authors read and approved the final manuscript.

## Supplementary Material

Additional file 1 Contains the supplemental figures. Click here for file

Additional file 2 Contains statistics of transcripts defined by the various steps. Click here for file

Additional file 3 The Emihu1plus version of the genome. Click here for file

Additional file 4 Contains the sequences of the “gold standard” genes. Click here for file

Additional file 5 A table of how the “gold standard” genes were derived and which were validated. Click here for file

Additional file 6 Contains examples of sequences that were found on the DNA level but not on the protein level. Click here for file

Additional file 7 A table of transcript annotations. Click here for file
