# Supplementary material for: Improving transcriptome construction in non-model organisms: integrating manual and automated gene definition in Emiliania huxleyi
Source: BMC Genomics. 2014 Feb 22;15:148. doi: 10.1186/1471-2164-15-148 (PMC4028052; doi:10.1186/1471-2164-15-148)
Supplement: Additional file 1 — Contains the supplemental figures. [file 1471-2164-15-148-S1.docx]

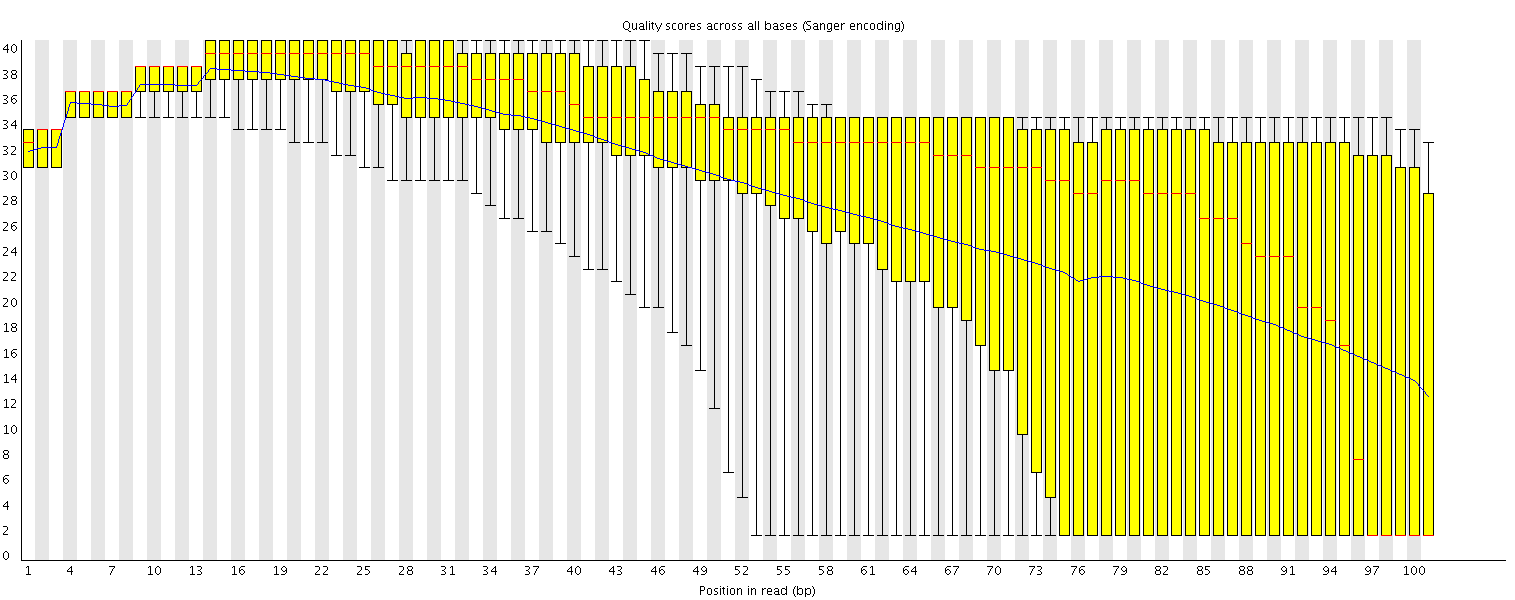


**Figure S1. Quality scores of RNAseq reads.** The graph was obtained using the FastQC software.

**Figure S2. Comparison of our transcripts and the JGI ‘Best model’ transcripts.**


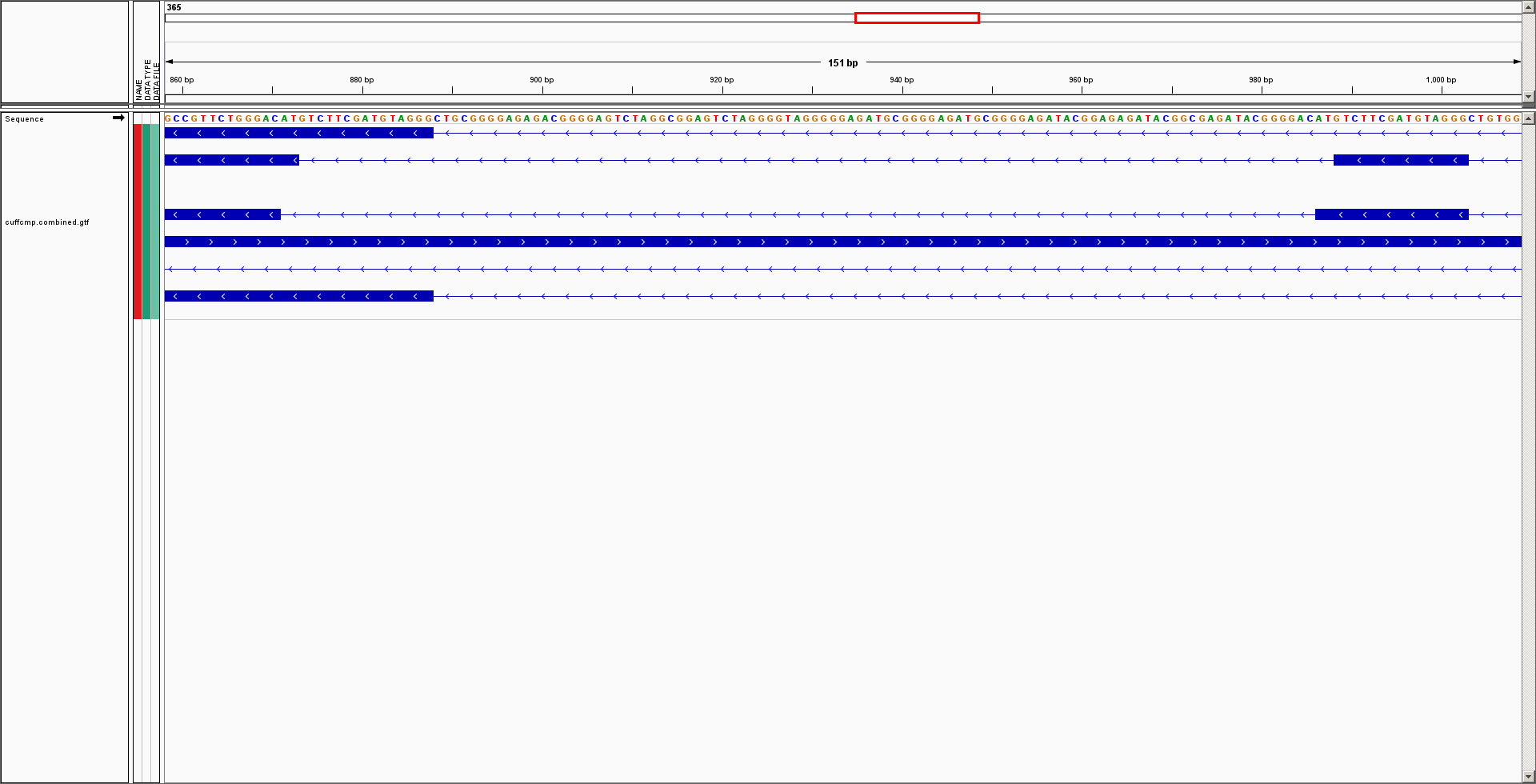


**Figure S3. An example of a poorly defined splice junction.**


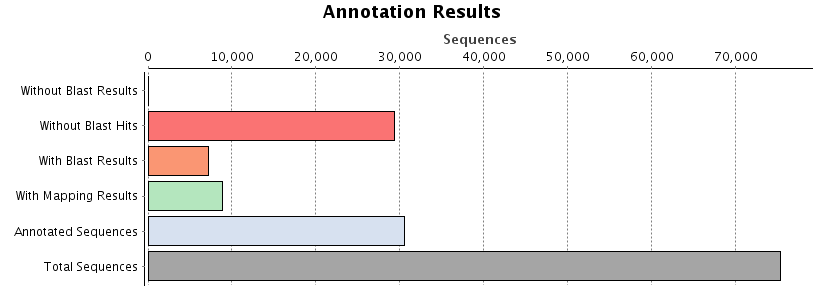


**Figure S4. Blast2Go annotation summary.**


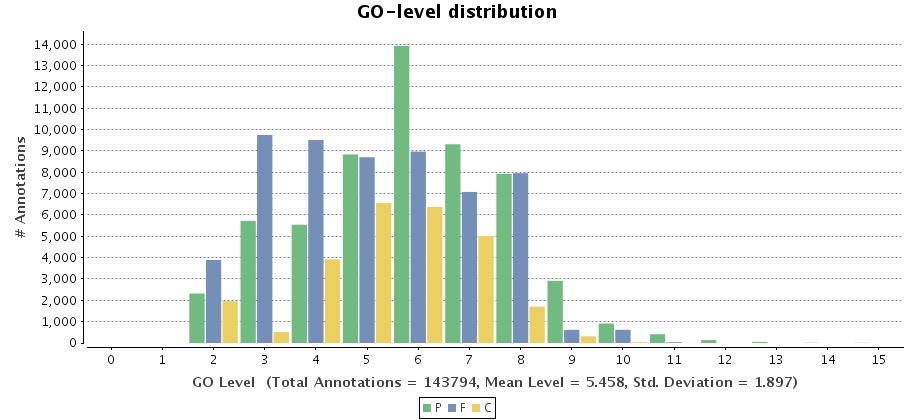


**Figure S5. Distribution of Gene Ontology levels taken from Blast2GO.**


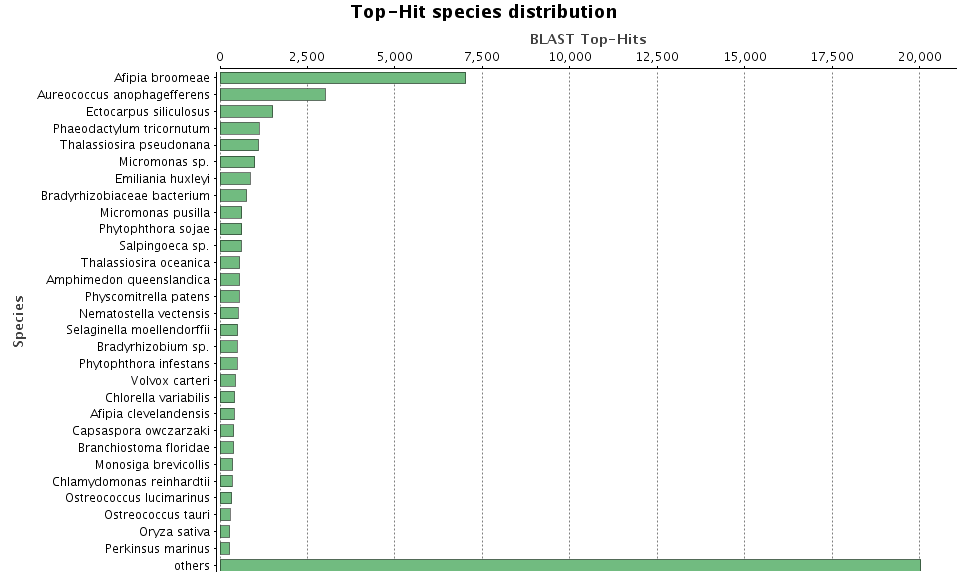


**Figure S6. Species distribution of top Blast hits in Blast2GO.**


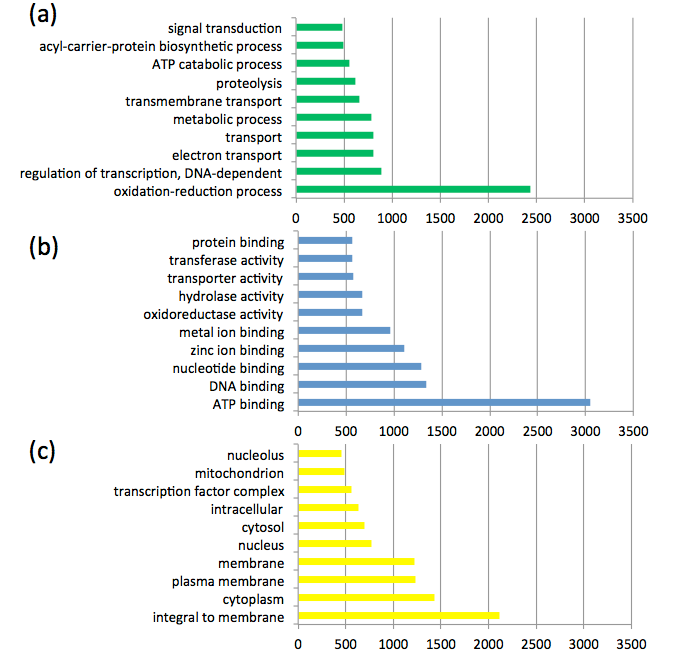


**Figure S7. Blast2GO Gene Ontology categories.** Number of genes per 10 top annotations A. Biological Process B. Molecular Function C. Cellular Component


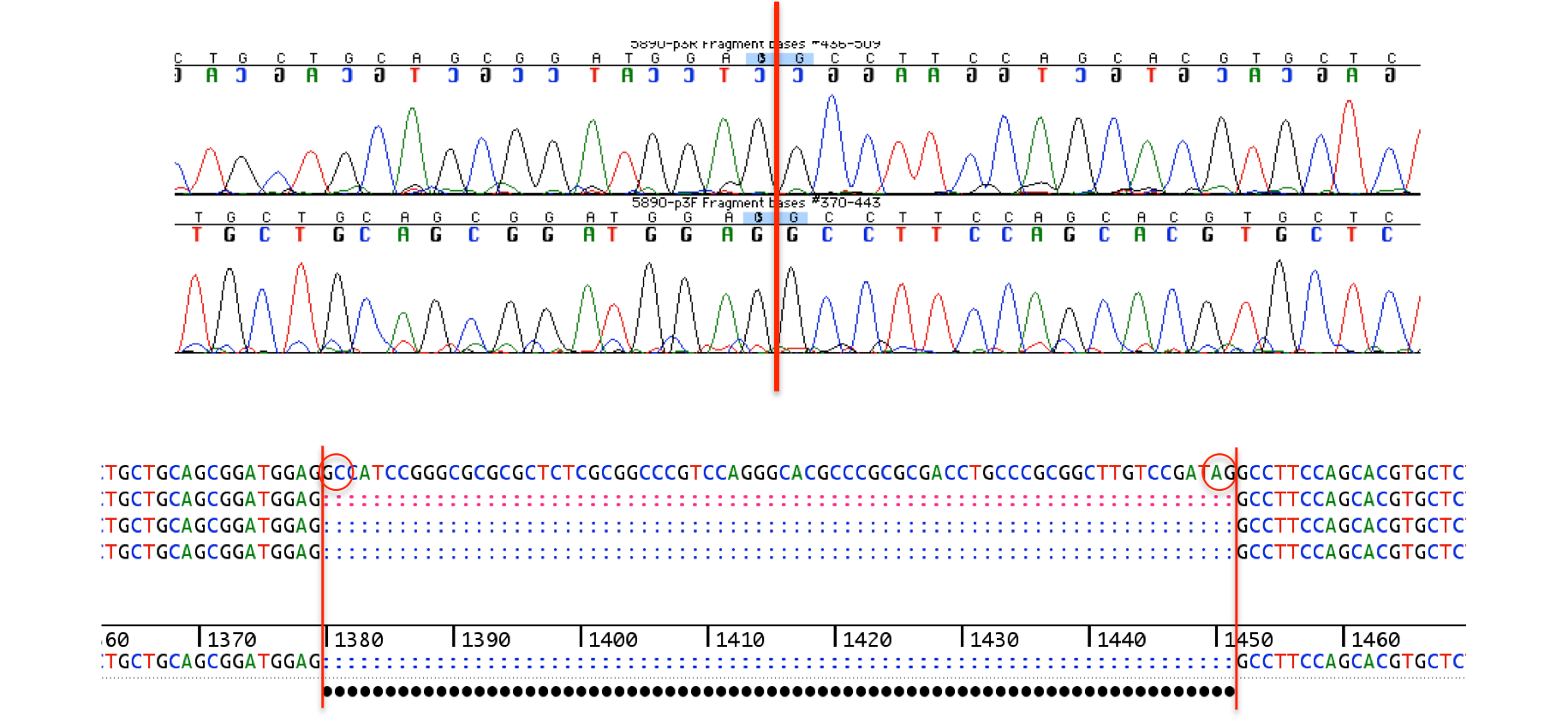


**Figure S8. Example of Sanger validated junction**. Transcript number 61 was chosen for validation and reverse transcriptase PCR of a region covering five junctions was performed. An example of one GC:AG junction is shown. The splice junction is indicated by red bars, and the genomic flanking bases are encircled in red. In the bottom panel, the top sequence with no spacer is the genomic sequence. The second sequence, with the red spacer, is the manually built one, and the sequences with the blue spacers are the Sanger sequences in both directions (corresponding chromatograms shown above). The junction cannot be mapped in any other position, unless a completely non-canonical splicing pattern is used; on the 5’ end, the bases AG can be moved to the other side of the junction, but that creates an AG:AT junction, and on the 3’ end, the bases GCC can be moved to the other side of the junction, but that creates an AT:CC junction. Primers used for validation are as follows: Transcript 61: 5’ CGTGATGCAGCTCTTCGG and 3’ CATCACCGAGTCCTTGTTGC; Transcript 37: 5’ ACAGGCGACGCTTTTGTG and 3’ ATCTTGGACATGTCGCCGAT; and Transcript 57: 5’ TCAAGCACGCCGGTGAT and 3’ GATCAAACCGAGGGATCGC.


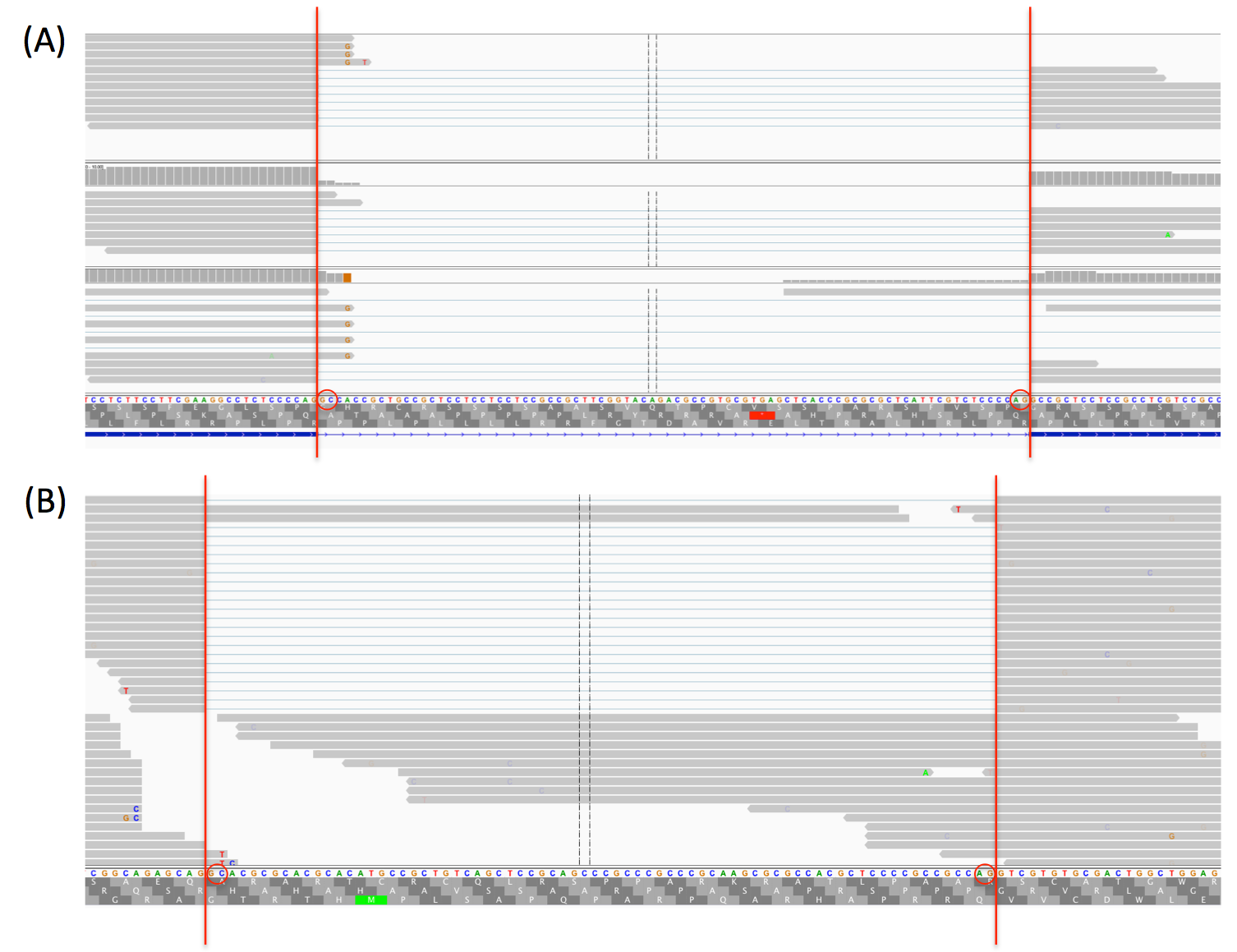


**Figure S9. Examples of manually inspected RNA-Seq read junctions.** 270 junctions were inspected manually, and two examples of GC:AG junctions are given above. The splice junctions are indicated by red bars, and the genomic flanking bases are encircled in red. In both cases only part of the reads that span the junction are shown. The sequences cannot be mapped in any other position, unless a completely non-canonical splicing pattern is used. In panel A, on the 5’ end, the bases TCTCCCCAG can be moved to the other side of the junction, but that creates a TC:CG junction, and on the 3’ end, the bases GCC can be moved to the other side of the junction, but that creates an AC:CC junction. In panel B, on the 5’ end, the bases CAG can be moved to the other side of the junction, but that creates a CA:CG junction, and on the 3’ end, the base G can be moved to the other side of the junction, but that creates a CA:GG junction. Extensive intronic readthrough can be seen in panel B, while minor readthrough is seen in panel A.
