# Supplementary material for: Improving transcriptome construction in non-model organisms: integrating manual and automated gene definition in Emiliania huxleyi
Source: BMC Genomics. 2014 Feb 22;15:148. doi: 10.1186/1471-2164-15-148 (PMC4028052; doi:10.1186/1471-2164-15-148)
Supplement: Additional file 3 — The Emihu1plus version of the genome. [file 1471-2164-15-148-S3.docx]

Additional File 3: Emihu1plus sequence is available at:

http://incpm-2.weizmann.ac.il/bioinfo/Esti/txpaper/Additional_file_1.txt
