# Supplementary material for: Improving transcriptome construction in non-model organisms: integrating manual and automated gene definition in Emiliania huxleyi
Source: BMC Genomics. 2014 Feb 22;15:148. doi: 10.1186/1471-2164-15-148 (PMC4028052; doi:10.1186/1471-2164-15-148)
Supplement: Additional file 6 — Contains examples of sequences that were found on the DNA level but not on the protein level. [file 1471-2164-15-148-S6.docx]

Examples of sequences that were found on the DNA level, but not on the protein level.

Example 1: Readthrough and splicing issues

Transcript 38: Compared to genome sequence (Emihu1, Scaffold 11). Introns in bold blue. Compared to transcripts: CL13218Contig1 has no introns (readthrough), while 11512 has an extended first intron. Neither was constructed correctly by automatic means, and therefore, the translations were wrong, and the gene was found only on the DNA level, but not on the protein level.


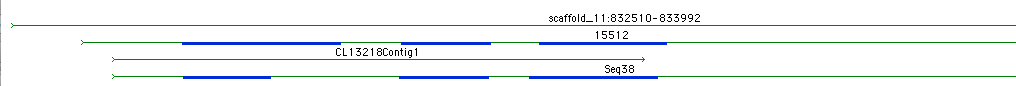


Example 2: reading frame issues

Transcript 13 – “gold standard” compared to CL3749Contig1 on the protein level. The script used to translate looked at all reading frames longer than 100 amino acids, and took the longest ORF from stop to stop, ignoring the Methionine positions. In this case the longest ORF is in frame 3 forward, with 335 amino acids, while the correct frame is in frame 3 reverse with 334 amino acids.

M = methionine, * = stop codon

ORF = M to stop, rest of frame not in yellow: stop to M or stop to stop

5'3' Frame 1

X X X P G S G Q R A R A A A S A T G V P F Y L F I Y A M L D L E F S I S Y W T G S P V G C S V T * A R R R P Y T N Y D Q L L F M I F S P P R A R S P S G R H V P S A L S I K M P T P K F H E K C S W C A S C S S A P K A R * P L C A R L V D V R E K A S Q * A A A * G * Q R V S A G P R T T P P T L P T Q N S I G L A Y T H A M A A G A L K A W W N L W I H L Y R S G R W S A R W V T * N H T S I V T T C T A K H A S S L G K D S G V S H A P E P P P R R C S C H A G I V A N Q E K R R I L D S P T A T A R V H * S H V K M E R W P C V L Y R C A S G W Q S R S E Q S P T A T T R * A D S A L Q P I K T A V S S C A G S R Y G R S A A G H M A S S A R V R W P H M G R L T N R T Q K N A T V A V M R C E A L P A A R A P R G A A A G G A R S A R N S G S S S T G * G R G L L V A M K S V G E S P G P F W A A S R T S

5'3' Frame 2

X X G P A A A S A R A Q R P A Q Q A F R F I Y L F M P C * T S N F L F P T G L G P L * A V A * R K Q E D G P I R I T I S F S S * S S R P P A R A A P P A G T C R A P C P S R C R R R S S T K S A R G A H R A P L R Q R R G S R C A P G S S T * G K R R A S R R R R R G D S A S A P A P E P R R R R C R R R T R S G W R T R M R W R R A P * R R G G T C G S T C T G A A G G A R G G * R R T T R R L S R R A P Q S T P A R W A R T A A C P T R P S R L R A A A A A T P G * S R T R R R G G Y W T A R R R P R A S T D P T * R W S G G P A S C T G A P P G G S P G A S R V L P R R R G R Q T A H C S P S R P R C R P A P G R G T A A A R P A T W P R V H A S D G R T W E D * Q T G R R R T R P W R * * G A R P C R R R E R H A E L L P A E L G P H E T L A P P P Q G K G G A C W S P * S L W V R V P G L F G P R R A P R

5'3' Frame 3 = 335 amino acids

X X A R Q R P A R A R S G Q R N R R S V L F I Y L C H A R P R I F Y F L L D W V P C R L * R D V S K K T A L Y E L R S A S L H D L L A P P R A Q P L R Q A R A E R P V H Q D A D A E V P R K V L V V R I V L L C A K G A V A A V R Q A R R R E G K G E P V G G G V G V T A R Q R R P Q N H A A D V A D A E L D R V G V H A C D G G G R L E G V V E L V D P L V Q E R Q V E R A V G D V E P H V D C H D V H R K A R Q L V G Q G Q R R V P R A R A A S A P L Q L P R R D S R E P G E E E D I G Q P D G D R A R P L I P R K D G A V A L R L V P V R L R V A V Q E R A E S Y R D D E V G R Q R T A A H Q D R G V V L R R V E V R P Q R G R P H G L E C T R Q M A A H G K T N K P D A E E R D R G G D E V R G L A G G E S A T R S C C R R S S V R T K L W L L L H R V R A G P A G R H E V C G * E S R A F L G R V A H L

3'5' Frame 1

P R C A T R P K K A R D S H P Q T S W R P A G P A L T L W R R S Q S F V R T E L R R Q Q L R V A L S P P A R P R T S S P P R S R S S A S G L L V F P C A A I * R V H S R P C G R P R C G R T S T R R R T T P R S * W A A V R C L P T S S S R * D S A R S W T A T R R R T G T R R R A T A P S L R G I S G R A R S P S G C P I S S S S P G S R L S R R G S C S G A E A A R A R G T R R C P C P T S W R A L R C T S * Q S T C G S T S P T A R S T C R S C T S G S T S S T T P S R R P P P S H A C T P T R S S S A S A T S A A W F W G R R * R A V T P T P P P T G S P F P S R R R A W R T A A T A P L A Q R S T M R T T S T F R G T S A S A S * W T G R S A R A C R R G C A R G G A R R S * R E A D R N S Y R A V F L L T S R Y S L Q G T Q S S R K * K I R G L A W H K * I N K T E R L L R W P L R A R A G R C R A X X

3'5' Frame 2 = 294 amino acids

R G A R R G P K R P G T L T H R L H G D Q Q A P P L P C G G G A R V S C G P S S A G S S S A W R S R R R Q G L A P H H R H G R V L L R P V C * S S H V R P S D A C T R G H V A G R A A A V P R P G A G R H R G L D G L Q C A V C L P R R R G R T L L A P G L P P G G A P V Q D A G P P L H L Y V G S V D A R G R R R A V Q Y P P L L L V R D Y P G V A A A A A R R R L G R V G H A A V L A Q R A G V L C G A R R D N R R V V L R H P P R A P P A A P V Q V D P Q V P P R L Q G A R R H R M R V R Q P D R V L R R Q R R R R G S G A G A D A L S P L R R R L L A R L F P H V D E P G A Q R L P R L W R R G A R C A P R A L F V E L R R R H L D G Q G A R H V P A G G A A R A G G R E D H E E K L I V I R I G P S S C L R H A T A Y R G P S P V G N R K F E V * H G I N K * I K R N A C C A G R C A R A L A A A G P X A

3'5' Frame 3 = 334 amino acids

E V R D A A Q K G P G L S P T D F M A T S R P R P Y P V E E E P E F R A D R A P P A A A P R G A L A A G K A S H L I T A T V A F F C V R F V S L P M C G H L T R A L E A M W P A A L R P Y L D P A Q D D T A V L M G C S A L S A Y L V V A V G L C S L L D C H P E A H R Y K T Q G H R S I F T W D Q W T R A V A V G L S N I L L F S W F A T I P A W Q L Q R R G G G S G A W D T P L S L P N E L A C F A V H V V T I D V W F Y V T H R A L H L P L L Y K W I H K F H H A F K A P A A I A C V Y A N P I E F C V G N V G G V V L G P A L T R C H P Y A A A Y W L A F S L T S T S L A H S G Y R A F G A E E H D A H H E H F S W N F G V G I L M D R A L G T C L P E G L R A R G G E K I M K R S * S * F V * G R L L A Y V T L Q P T G D P V Q * E I E N S R S S M A * I N K * N G T P V A L A A A R A R W P L P G Q X
